# Analysis of Cardiovascular Risk Factors in Patients with Psoriasis: A Cross-Sectional Study

**DOI:** 10.31138/mjr.300125.pac

**Published:** 2025-07-17

**Authors:** Barbara S. Kahlow, Ana Paula Beckhauser, Thelma L. Skare, Renato Nisihara

**Affiliations:** 1 Department of Medicine, Mackenzie Evangelical School of Medicine Paraná, Curitiba, Paraná, Brazil;; 2 Department of Medicine, Positivo University, Curitiba, Paraná, Brazil

**Keywords:** arthritic psoriasis, psoriasis, atherosclerosis, carotid atherosclerosis, inflammation

## Abstract

**Objectives:**

This study aimed to evaluate and compare atherosclerotic risk factors and carotid intima-media thickness (cIMT) between psoriasis patients with and without arthritis.

**Methods:**

Data on demographics, clinical characteristics, comorbidities, and treatments were collected in the medical charts. Laboratory assessments, including lipid profile and fasting glucose, were performed, along with cIMT measurements via ultrasound.

**Results:**

A total of 127 participants were analysed, including 49 controls and 78 psoriasis patients (47 with psoriatic arthritis and 31 without). Psoriasis patients exhibited a higher frequency of diabetes (OR=2.3; 95% CI=1.009–5.08) and hypertension (OR=6.8; 95% CI=1.7–30.8) compared to controls. Additionally, cIMT values were significantly elevated in psoriasis patients compared to controls (median 0.68 mm vs. 0.57 mm, p=0.001). However, no significant differences in traditional atherosclerotic risk factors or cIMT measurements were observed between psoriasis patients with and without arthritis (all p>0.05).

**Conclusion:**

Psoriasis patients, regardless of arthritis status, exhibit increased atherosclerotic risk compared to controls. However, in this sample, it was not possible to prove that the presence of arthritis further exacerbates this risk.

## INTRODUCTION

Psoriasis is a widespread chronic inflammatory skin disorder that is now widely recognised for its association with an increased risk of atherosclerosis and its related complications.^1^ Patients with psoriasis have been shown to exhibit a 1.78-fold higher likelihood of cerebrovascular events,^1^ a 1.98-fold greater risk of peripheral vascular disease,^1^ and an elevated probability of developing myocardial infarction: 1.72 times more in mild psoriasis cases and 2.01 times more in severe cases compared to control populations.^2^ Mehta et al.^3^ further highlighted that individuals with severe psoriasis face an additional 6.2% risk of experiencing a major cardiovascular event within 10 years, relative to the general population.

In this context, the higher prevalence of comorbidities, such as diabetes mellitus (DM), obesity, and hypertension, in individuals with psoriasis has been noted as a contributing factor to the increased cardiovascular risk.^1–3^ Furthermore, chronic inflammation, a hallmark of psoriasis, is also believed to play a pivotal role in these complications. Atherosclerosis is considered a chronic inflammatory condition of the arterial wall, susceptible to modulation by concurrent inflammatory and immune responses, as observed in diseases like RA, spondylarthritis, and lupus.^4^ Elevated levels of cytokines such as TNF-alpha, IL-6, IL-1, and IL-17 in inflammatory diseases have been associated with mechanisms that drive atherogenesis and atherothrombosis.^5^

Psoriatic arthritis (PsA) affects up to 30% of individuals with psoriasis, manifesting as pain, stiffness, swelling, and tenderness in the joints, as well as inflammation in adjacent tendons and ligaments.^6^ This additional inflammatory burden raises the hypothesis that patients with psoriatic arthritis might have a worse prognosis from an atherosclerotic standpoint compared to those with psoriasis limited to the skin.

Although several studies have addressed the prevalence of traditional atherosclerotic risk factors in psoriasis patients with and without arthritis, only a limited number have compared the occurrence of atherosclerosis and cardiovascular events in these two subsets. To our knowledge, no such studies have focused on the Brazilian population.

In this study, we analysed a sample of Brazilian psoriasis patients with and without arthritis, comparing their atherosclerotic risk factors and carotid intima-media thickness (cIMT) measurements. Our objective was to determine whether psoriatic arthritis imposes an additional burden on the atherosclerotic process.

## MATERIAL AND METHODS

### Study design

Cross-sectional study.

### Ethical issues

This study was approved by the Committee of Ethics in Research from Faculdade Evangélica Mackenzie do Paraná under protocol 4.166.908, in 21/06/2020. All participants signed consent.

### Participants

A convenience sample was analysed, including all patients with psoriasis and psoriatic arthritis who attended regular consultations over the course of one year at a Dermatology and Rheumatology Clinic in a single Public University Hospital. Patients were included in the study based on the order of their appointments and their willingness to participate. Inclusion criteria required patients to be older than 18 years and have a diagnosis of psoriasis confirmed by biopsy or a certified dermatologist. Patients with psoriatic arthritis were required to meet the CASPAR classification criteria.^7^ Exclusion criteria included pregnant women, individuals with renal failure, other inflammatory diseases, or neoplasms. Self-declared healthy individuals accompanying patients to their consultations were included as controls.

### Data Collection

Data collection included epidemiological information (age, sex, self-declared ethnic background, tobacco, and alcohol use) and clinical data (psoriasis subtype, scalp and nail involvement, psoriatic arthritis subtype, treatments used, and comorbidities). These data were obtained through chart review and direct patient questioning.

Weight and height measurements were collected to calculate the body mass index (BMI), along with abdominal circumference, which was obtained through a physical examination. BMI was calculated by dividing weight in kilograms by height in meters squared. Waist circumference was measured at the midpoint between the last rib and the iliac crest with the patient standing. Blood pressure was measured with the patient seated after a 10-minute rest using an aneroid sphygmomanometer.

Laboratory tests, including total cholesterol, HDL and LDL cholesterol, triglycerides, and fasting glucose, were performed concurrently with data collection. These measurements were conducted after 12 hours of fasting using the enzymatic colorimetric method. The presence of metabolic syndrome was determined according to the criteria of the National Cholesterol Education Program’s Adult Treatment Panel III (NCEPATP III).^8^

The extent of skin disease was evaluated using the Psoriasis Area and Severity Index (PASI), which takes into account the severity of erythema, induration, and desquamation, as well as the percentage of the affected area. The PASI score ranges from 0 to 72, with higher scores indicating more severe disease.^9^

*Measurement of cIMT (Carotid Intima-Media Thickness)* The cIMT was measured by a single investigator who was blinded to the clinical data, using an Esaote® ultra-sound system (model MyLab40) with high resolution, operating in B-mode with an 18 MHz linear transducer. The examination was conducted in a quiet, air-conditioned room (22°C), with patients in a supine position, their necks extended and rotated 45 degrees away from the side being examined. The carotid artery was observed in both transverse and longitudinal planes, and measurements were taken at a distance of 10–20 mm from the carotid bifurcation in the distal vessel wall.^10^ A carotid artery plaque was defined as a focal thickening that protrudes into the arterial lumen by at least 0.5 mm, or by 50% relative to the adjacent intima-media thickness (IMT), or presents a total thickness greater than 1.5 mm, measured from the media-adventitia interface to the intima-lumen interface.^10^ Both sides of the carotid artery were examined, and for statistical purposes, the highest measurement was considered.

### Statistical Analyses

Data were analysed using frequency and contingency tables. The Shapiro-Wilk test was applied to assess data distribution, with central tendency expressed as mean and standard deviation (SD) for parametric data or median and inter-quartile range (IQR) for non-parametric data. Fisher’s exact test and chi-squared tests were used to compare categorical variables, while unpaired t-tests and Mann-Whitney tests were used to compare continuous variables. Statistical significance was set at 5%. Correlation studies were done using Spearman test. All analyses were conducted using GraphPad Prism version 8.0.0 for Windows (GraphPad Software, San Diego, California, USA; www.graphpad.com).

## RESULTS

The studied sample had 127 individuals: 49 controls and 78 with psoriasis (47 with psoriatic arthritis - and 31 with skin disease only). **Table 1** shows the description of psoriasis sample that had middle aged individuals, mainly Euro descendants and with plaque psoriasis.

**Table 1.** Description of studied sample patients with psoriasis (n=78).

Median age - (IQR) years	49 (44–57)
Sex: Females/males	55.1%/44.9%
Ethnic background	Euro descendants - 84.6%
	Afro descendants - 15.3%
Smokers	24.3%
Psoriasis subtype	Plaque - 79.3%
	Palm-plantar - 11.1%
	Others -12.6%
Nail involvement	44.4%
Scalp involvement	65.0%
Median disease duration (IQR) months	108.0 (48.0–219.0)
Median PASI – (IQR)	2.8 (0.9 – 6.0)
Median DAPSA – (IQR) (*)	16.0 (8.0–22.1)
Arthritis subtypes (*)	Axial involvement - 25.5%
	Oligoarticular - 31.9%
	Polyarticular - 63.8%
	Distal interphalangeal involvement - 10.6%
Treatment	Methotrexate - 33.3%
	Leflunomide - 6.4%
	Biologicals - 43.5%
	Anti-TNF alpha - 28.2%
	Anti-IL-17 - 10.2%
	Anti-IL-12/23 - 5.1%

IQR: interquartile range; DAPSA: Disease Activity in Psoriatic Arthritis; PASI: Psoriasis Area Severity Index; IL: interleukin; TNF: tumour necrosis factor; *refers to patients with arthritis only.

Controls were paired with psoriasis patients for age (p=0.12), sex (p=0.36) and ethnic background (p=0.99). **Table 2** compares controls with psoriasis patients— with and without arthritis—and shows that those with psoriasis had higher rates of tobacco exposure and diabetes mellitus than controls. In addition, arterial hypertension was more prevalent among patients with psoriatic arthritis than in controls, while the prevalence of metabolic syndrome and BMI were similar across the groups. Notably, PASI scores were higher in patients with cutaneous psoriasis only compared to those with psoriatic arthritis.

**Table 2.** Comparison of psoriasis patients (with and without arthritis) and controls.

	**Controls**	**Total Pso**	**P** **Total Pso vs controls**	**Pso A**	**P** **Total Pso A vs controls**	**Pso (skin only)**	**P** **Pso A vs Pso (skin only)**
n	49	78	-	47	-	31	-
Tobacco exposure (current and ex-smokers)	12.2%	44.8%	0.002 *	44.6%	0.005 **	45.1%	0.86
Median PASI (IQR) -	-	2.8 (0.9–6.0)	-	1.8 (0.0–4.2)	-	4.0 (2.1–7.6)	0.01
Dyslipidemia -	41.6%	55.1%	0.24	78.3%	0.10	45.1%	0.15
Diabetes mellitus -	4.7%	25.6%	**0.005 ^#^**	23.4%	**0.01^##^**	29.7%	0.57
Arterial Hypertension -	32.4%	52.5%	**0.04 ^§^**	53.1%	**0.05^§§^**	51.6%	0.89
Metabolic Syndrome -	28.5%	34.6%	0.31	34.0%	0.54	35.4%	0. 89
Median BMI (SD) – kg/m^2^	27.0 (24.3–32.0)	29.8 (26.4–33.7)	0.22	31.3 (27.0–36.7)	0.09	27.7 (25.0–31.5)	0.06
Mean abdominal circumference (SD)- cm	100.6 (17.9)	104.2 (13.7)	0.30	106 (13.4)	0.15	101.4 (13.8)	0.14
Median systolic blood pressure (IQR) - mm Hg	120 (120–130)	120 (120–140)	0.56	120 (120–132)	0.81	125 (120–140)	0.59
Median diastolic blood pressure (IQR)- mm Hg	80 (80–80)	80 (80–90)	0.72	80 (70–80)	0.37	80 (80–90)	0.25
Median HDL cholesterol (IQR)- mg/dL	45.0 (37.5–53.2)	47.0 (40.0–56.0)	0.71	47.0 (40.0–57.0)	0.50	44.5 (40.2–53.2)	0.49
Median LDL cholesterol (IQR) – mg/dL	126.6 (105.0–148.45)	119.0 (92.2–142.0)	0.09	118.0 (92.1–138.0)	**0.04**	123.5 (96.0–152.1)	0.18
Median triglycerides (IQR) – mg/dL	138,0 (102.1–179.0)	141.0 (106.0–201.0)	0.61	137.0 (106.0–193.0)	0.71	149.0 (97.4–207.5)	0.72
Median glycemia (IQR) mg/dL	93.0 (82.0–97.0)	93.5 (85.0–110.5)	0.30	95.0 (87.7–104.0)	0.26	91.0 (84.1–116.5)	0.70
Median hemoglobin A1c (IQR)-%	5.8 (5.5–5.9)	5.5 (5.3–6.0)	0.32	5.5 (5.3–5.8)	0.08	5.6 (5.2–6.4)	0.54
Median ESR (IQR) - mm	11.0 (9.0–25.0)	17.5 (8.0–31.0)	0.54	18.0 (8.0–37.0)	0.51	15.0 (10.0–30.0)	0.68
Median CRP (IQR)- mg/dL	0.99 (0.4–1.4)	2.2 (0.8–5.0)	**0.04**	2.1 (0.7–5.0)	0.09	3.1 (1.0–6.5)	0.33

* OR: 4.3; 95%CI: 1.6 to 11.2;

** OR: 4,3; 95%CI: 1.5 to 12.3;

*** OR: 4,3 95%CI: 1.5 to 14.3;

# OR: 6.2; 95%CI: 1.7 to 30.8;

## OR: 6.1; 95% CI: 1.4 to 28.6;

### OR: 8.1;95%CI: 1.7 to 39.2);

§ OR: 2.3; 95%CI: 1.009 to 5.);

§§ OR: 2.36; 95%CI: 0.96 to 5.7.

n: number; SD: standard deviation; IQR: interquartile range; OR: odds ratio; PASI: Psoriasis Area Severity Index; BMI: body mass index; HDL: High density lipoprotein; LDL: low density lipoprotein, ESR: erythrocyte sedimentation rate; CRP: C reactive protein; Pso: psoriasis; Pso A: psoriatic arthritis.

**Figure 1.**
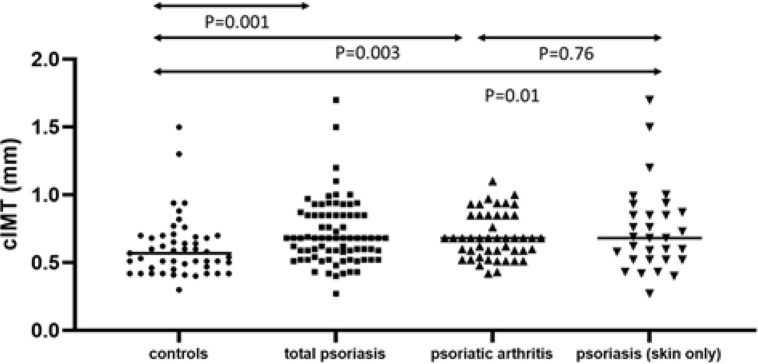
Differences in cIMT found between psoriasis patients with and without arthritis.

The comparison of carotid intima-media thickness (cIMT) between controls and the entire psoriasis cohort revealed a significantly higher median cIMT in psoriasis patients. This pattern persisted when controls were compared separately with patients with psoriatic arthritis and with those having only skin involvement. However, no significant differences in cIMT were found between psoriasis patients with and without arthritis (**Figure 1**). In this sample, neither patients nor controls exhibited atherosclerotic plaques.

In the psoriasis group (with and without arthritis) 5 patients have had myocardial infarction (2 or 4.2% in arthritis patients and 3 or 10.7% in patients with skin only); in the control group only one (p=0.40).

**Table 3** shows the cIMT values according to the studied variables in the 3 groups. In this table it is possible to see that the traditional atherosclerotic risk factor was associated with increased cIMT in patients with cutaneous psoriasis only, but not in those with psoriatic arthritis.

**Table 3.** cIMT studies according to the variables.

Association Studies*	**Total psoriasis sample**			**Psoriatic arthritis**			**Psoriasis (skin only)**		
	Positive	Negative	p	Positive	Negative	P	Positive	Negative	P
Female sex	0.68 (0.52–0.85)	0.68 (0.59–0.85)	0.47	0.68 (0.54–0.85)	0.68 (0.59–0.85)	0.64	0.64 (0.52–0.86)	0.69 (0.52–0.94)	0.62
Tobacco exposure	0.68 (0.59–0.85)	0.68 (0.52–0.68)	0.55	0.68 (0.55–0.89)	0.68 (0.57–0.85)	0.80	0.70 (0.59–0.87)	0.68 (0.47–0.90)	0.57
Hypertension	0.68 (0.59–0.93)	0.62 (0.52–0.74)	**0.01**	0.69 (0.56–0.85)	0.68 (0.57–0.72)	0.61	0.80 (0.63–0.99)	0.52 (0.43–0.76)	**0.002**
Diabetes mellitus	0.72 (0.62–0.93)	0.65 (0.52–0.85)	**0.03**	0.68 (0.59–0.94)	0.68 (0.55–0.82)	0.27	0.76 (0.68–0.96)	0.60 (0.49–0.85)	0.09
Dyslipidaemia	0.68 (0.59–0.85)	0.68 (0.52–0.76)	**0.05**	0.62 (0.53–0.85)	0.68 (0.59–0.78)	0.65	0.80 (0.68–1.05)	0.52 (0.43–0.76)	**0.001**
Metabolic syndrome	0.69 (0.60–0.94)	0.68 (0.52–0.85)	**0.02**	0.68 (0.59–0.91)	0.68 (0.54–0.76)	0.38	0.76 (0.68–1.2)	0.59 (0.45–0.85)	**0.02**
**Correlation studies**	**R**	**95%CI**	**p**	**R**	**95%CI**	**p**	**R**	**95%CI**	**P**
Age	0.18	–0.04 to 0.39	0.09	0.01	–0.27 to 0.31	0.90	0.41	0.06 to 0.67	**0.02**
Disease duration	–0.19	–0.43 to 0.06	0.13	–0.17	–0.46 to 0.14)	0.27	–0.34	–0.70 to 0.15	0.15
BMI	0.17	–0.06 to 0.38	0.13	0.25	–0.04 to 0.51	0.08	0.13	–0.24 to 0.47	0.47
DAPSA	-	-	-	–0.05	–0.43 to 0.33	0.76	-	-	-
PASI	0.11	–0.14 to 0,35	0.38	0.01	–0.34 to 0.37	0.91	0.14	–0.22 to 0.48	0.42
ESR	0.19	–0.04 to 0.40	0.09	0.25	–0.04 to 0.50	0.08	0.12	–0.27 to 0.49	0.52
CRP	0.13	–0.10 to 0.36	0,25	0.09	–0.20 to 0.38	0.51	0.21	–0.18 to 0.56	0.27

CI: confidence interval; PASI: Psoriasis Area Severity Index; BMI: body mass index; DAPSA: Disease Activity in Psoriatic Arthritis; ESR: erythrocyte sedimentation rate; CRP: C reactive protein.

* all values in median and interquartile range.

## DISCUSSION

This study aimed to compare the cardiovascular risk in psoriasis patients—with and without arthritis—using carotid intima-media thickness (cIMT) as a predictive marker.^11^ cIMT, a non-invasive ultrasound-based measurement of the intimal and medial layers of the carotid artery wall, is widely recognised as a reliable indicator of subclinical atherosclerosis and cardiovascular risk. The present findings demonstrate that patients with psoriasis exhibit an elevated risk of atherosclerotic vascular events, as evidenced by higher cIMT values compared to the general population. Notably, no significant differences in cIMT were detected between psoriasis patients with and without arthritis.

It is important to emphasise that traditional atherosclerotic risk factors appear to be more closely associated with increased cIMT in patients presenting with isolated cutaneous involvement. This finding may reflect differences in management approaches between dermatology and rheumatology, with the latter potentially implementing more intensive cardiovascular risk prevention strategies. Although the absence of significant differences in cIMT between the groups could suggest that the additional articular inflammatory burden does not further aggravate atherosclerotic risk, the heterogeneity in treatment regimens represents a potential confounding factor. Moreover, it is well established that patients with other inflammatory diseases lacking cutaneous manifestations, such as rheumatoid arthritis^12,13^ and ankylosing spondylitis,^14^ exhibit an increased risk of cardiovascular events. This supports the notion that joint inflammation alone may contribute to accelerated atherosclerosis. Conversely, inflammatory conditions confined to the skin, such as atopic dermatitis, are not typically associated with an increased cardiovascular risk.^15^ Despite these distinctions, psoriasis, whether accompanied by arthritis or not, shares a common inflammatory milieu, with skin involvement representing one aspect of a systemic autoimmune and autoinflammatory disorder, while joint involvement constitutes another clinical facet. In this context, Chimeneti et al.^16^ have proposed the term “systemic psoriatic disease” to emphasise the heterogeneous clinical manifestations of psoriasis underpinned by shared etiopathogenic mechanisms.

The European Alliance of Associations for Rheumatology (EULAR) advocates applying a 1.5 multiplication factor to cardiovascular risk scores in patients with rheumatoid arthritis, and this adjustment should similarly be extended to those with psoriatic arthritis.^17^ In fact, Kaleta et al.^17^ suggest that the severity of subclinical cardiovascular disease in psoriatic arthritis may even exceed that observed in rheumatoid arthritis.

The prevalence of atherosclerotic traditional risk factor between psoriasis patients with and without arthritis were similar. The only significant difference observed was a tendency to a higher BMI in patients with arthritis, potentially explained by reduced physical activity due to pain and joint inflammation, which may predispose to weight gain. Interestingly, PASI scores were higher in patients without arthritis. This finding can be attributed to the fact that our study was conducted in a tertiary care centre, where severe skin disease cases are referred. Conversely, arthritis patients are referred regardless of skin severity, while milder skin cases are often managed in primary care. This referral bias may have introduced a confounding factor, as the elevated PASI in patients without arthritis could offset the additional inflammation associated with arthritis.

Contrary to our findings, a study by Ragner et al.,^18^ comparing cardiovascular risk factors in patients with rheumatoid arthritis, psoriatic arthritis, and skin-only psoriasis, found that diabetes, hypertension, and hyperlipidaemia were more prevalent in psoriatic arthritis patients. However, this study did not focus on cardiovascular outcomes, and it is well established that traditional risk factors alone do not fully account for the atherosclerotic risk associated with psoriasis. Supporting this notion, Husted et al.^19^ observed a higher prevalence of traditional cardiovascular risk factors and myocardial infarction in psoriatic arthritis patients compared to those with skin-only psoriasis. In contrast, a study by Egeberg et al.^20^ reviewed the prevalence of myocardial infarction in psoriasis patients and reported a slightly higher risk in those with psoriasis and psoriatic arthritis, predominantly in patients without arthritis. Our findings similarly showed a lower frequency of myocardial infarction in arthritis patients; however, the small number of events and the limited sample size in our study preclude definitive conclusions.

Interestingly, Eder et al.^21^ found increased cIMT values in psoriatic arthritis patients compared to skin-only psoriasis patients. This discrepancy could be attributed to genetic variations, as alleles such as HLA-B*13:02 and HLA-C*06:02 have been linked to more severe atherosclerosis and elevated erythrocyte sedimentation rates, even after adjusting for cardiovascular risk factors.^21^ Genetic differences in the studied populations could explain the inconsistencies observed across the various studies.

This study is limited by its cross-sectional design and the relatively small sample size. A larger prospective cohort would enable a more robust investigation, allowing the analysis of cIMT variations across different psoriatic arthritis subtypes, which would provide valuable insights given the heterogeneity of this condition. Other limitations are the lack of familial cardiovascular history and the study of possible influence of cumulative glucocorticoid doses. The findings of this study should be interpreted with caution, as multivariate statistical analyses were not conducted. This decision was based on the authors’ evaluation that the sample size did not meet the minimum requirements to ensure the reliability and validity of multivariate modeling, which could lead to unstable estimates and potential overfitting. Additionally, body composition was not assessed in the present study; only BMI was measured, which is an inadequate surrogate. Kavadichanda et al.^22^ have noted that ectopic adipose deposition in skeletal muscle, secondary to sarcopenia, may influence cardiovascular risk.

## CONCLUSION

In this sample, Brazilian patients with psoriasis exhibited a higher prevalence of diabetes, hypertension, and elevated cIMT values compared to controls. cIMT and frequency of traditional atherosclerotic risk factors were similar in patients with and without arthritis.

## Data Availability

Data cannot be shared publicly due to ethical reasons as it contains individual patient details (confidential data). Data are available from the correspondence author (Dr. Renato Nisihara, renatonisihara@gmail.com) for researchers who meet the criteria for access to confidential data.
